# Could nanotechnology improve exercise performance? Evidence from animal studies

**DOI:** 10.1590/1414-431X2024e13360

**Published:** 2024-04-19

**Authors:** M.R. Lima, B.J. Moreira, R. Bertuzzi, A.E. Lima-Silva

**Affiliations:** 1 Grupo de Pesquisa em Desempenho Humano, Universidade Tecnológica Federal do Paraná, Curitiba, PR, Brasil; 2 Instituto de Física de São Carlos, Universidade de São Paulo, São Carlos, SP, Brasil; 3 Grupo de Estudos em Desempenho Aeróbio, Escola de Educação Física e Esporte, Universidade de São Paulo, São Paulo, SP, Brasil

**Keywords:** Nanotechnology, Exercise performance, Sport nutrition, Metabolism

## Abstract

This review provides the current state of knowledge regarding the use of nutritional nanocompounds on exercise performance. The reviewed studies used the following nanocompounds: resveratrol-loaded lipid nanoparticles, folic acid into layered hydroxide nanoparticle, redox-active nanoparticles with nitroxide radicals, and iron into liposomes. Most of these nutritional nanocompounds seem to improve performance in endurance exercise compared to the active compound in the non-nanoencapsulated form and/or placebo. Nutritional nanocompounds also induced the following physiological and metabolic alterations: 1) improved antioxidant activity and reduced oxidative stress; 2) reduction in inflammation status; 3) maintenance of muscle integrity; 4) improvement in mitochondrial function and quality; 5) enhanced glucose levels during exercise; 6) higher muscle and hepatic glycogen levels; and 7) increased serum and liver iron content. However, all the reviewed studies were conducted in animals (mice and rats). In conclusion, nutritional nanocompounds are a promising approach to improving exercise performance. As the studies using nutritional nanocompounds were all conducted in animals, further studies in humans are necessary to better understand the application of nutritional nanocompounds in sport and exercise science.

## Introduction

It has been widely demonstrated that nutritional supplements improve exercise performance (for a review, see Peeling at al. (1)). Nutritional supplementation is mostly used to improve physical capabilities and abilities (2), speed up recovery between workouts (3), improve antioxidant activity (4), reduce oxidative stress and inflammation (5,6), increase substrate availability (7), and enhance mitochondrial function and quality (8,9).

The majority of studies, however, has focused on investigating the conventional form of nutritional supplementation (i.e., supplements without nanoencapsulation or non-functionalized through nanotechnology). Conventional supplements are rapidly degraded in the gastrointestinal tract and/or largely metabolized by enterocytes (10). Consequently, conventional supplements might have low bioavailability (11,12), which will inevitably reduce their ergogenic potential. On the other hand, nutritional nanocompounds have the advantage of better solubility, dissipation (13), bioavailability (14), and absorption (15). Nanotechnology also allows manipulation of the chemical and physical properties of the nanocompound surface, which provides site-specific action (16), controlled release (17,18), a longer half-life (19), and reduced toxicity (20).

Nanocompounds are colloidal structures measured on a nanometer scale (1 to 100 nanometers) (21). Nanocompounds are categorized based on size and shape (rod, globular, conical, hollow, coiled, plane, cylindrical, or asymmetrical), interfacial properties (particle charge, lipophilicity, or hydrophilicity), and vehicle material (lipid or polymeric) (for a review, see Saeed et al. (21) and Zhou et al. (22)). Nanocompounds can be formulated via the double emulsion solvent evaporation, high shear homogenization, spray-drying, or nanoprecipitation techniques (23-25). The nanocompounds that were used as nutritional nano-supplements included polymeric nanoparticles (26,27), lipid nanoparticles (27,28), protein nanoparticles (29), layered double hydroxide nanoparticles (30), gold nanoparticles (31), liposomes (32), micelles (33), and dendrimers (34).

Although nutritional nanocompounds present several advantages over conventional nutritional supplementation, their potential as ergogenic aids has been much less explored. A better understanding of the benefits of nutritional nanocompounds in the context of exercise performance might provide insights into new strategies to optimize the action of nutritional supplements and improve exercise performance. Therefore, the present review was conducted to provide the current state of knowledge regarding the use of nutritional nanocompounds for improvement in exercise performance. A summary of the main structural characteristics and mechanisms of absorption of the nutritional nanocompounds is also provided. Data summarized in this review might assist future studies in investigating the potential of nutritional nanotechnology in the context of sports and physical activity. The Web of Science, Virtual Health Library (VHL), Scopus, and PubMed databases were utilized in the search (up to April 2023) using the following terms: nanotechnology* OR nanocomposite OR nanosupplement OR nanoparticles* OR liposome* OR nanocarrier* OR nanocapsule AND “nutrition supplements” AND sport OR exercise AND performance.

## Composition and structure of nanocompounds explored in exercise performance

There is an infinity of nanocarriers with different compositions and structures (35). However, the nanocarriers used in the reviewed studies linking nutritional nanocompounds and exercise performance were lipid nanosystems, redox-active nanoparticles, and layered double hydroxide nanoparticles.

**Figure 1:**
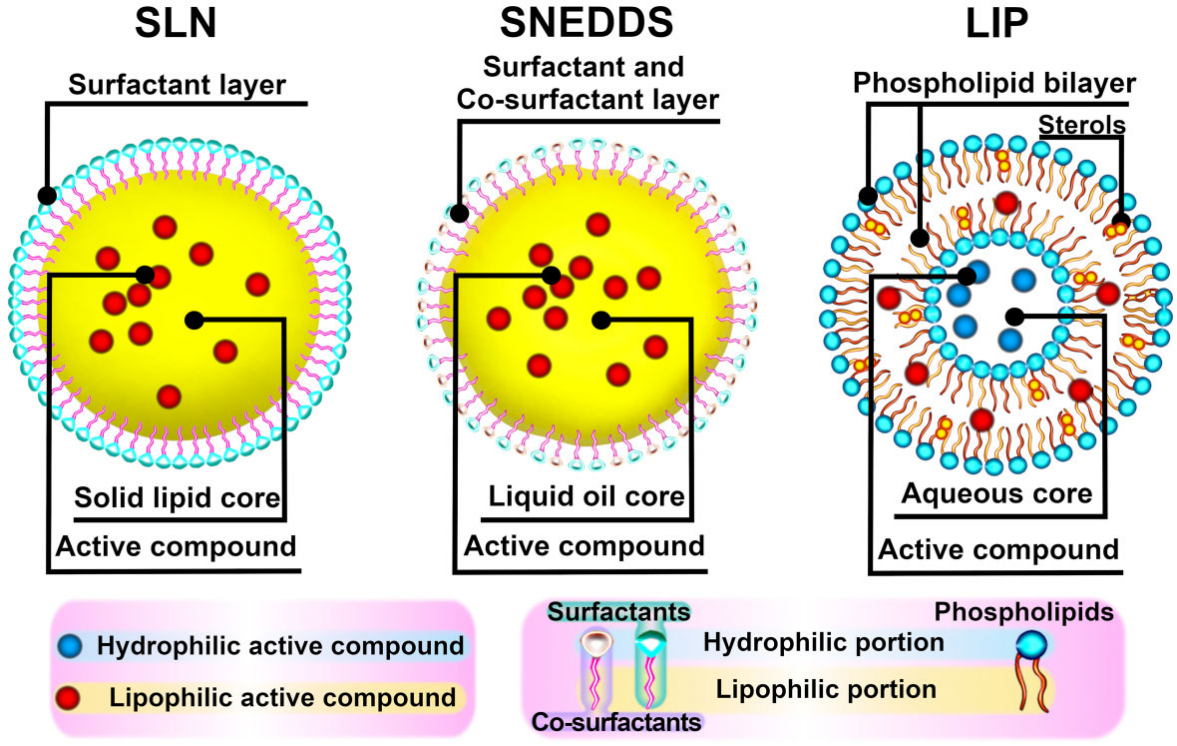
Schematic representation of the nanocarriers solid lipid nanoparticle (SLN), a self-nanoemulsifying drug delivery system (SNEDDS), and a liposome (LIP).

The lipid based nanosystems used in the reviewed studies were solid lipid nanoparticles (36,37), a self-nanoemulsifying drug delivery system (14), and liposomes (38). Solid lipid nanoparticles (Figure 1, left) are typically spherical particles coated with a surfactant layer that encapsulates the active compound into the solid lipid core (39). One advantage of solid lipid nanoparticles is that the nanocarrier toxicity is very low because it is composed of biological material, such as fatty acids or triglyceride mixtures (25,40,41).

The self-nanoemulsifying drug delivery system (Figure 1, middle) is a mixture of surfactants with natural or synthetic oils, such as coconut oil (42), methyl oleate, and ethyl oleate (43). This mixture spontaneously forms an oil-in-water emulsion composed of a membrane layer (surfactant and co-surfactant layer) and a liquid oil core, in which the active compound is allocated in the latter (41,44). The use of co-surfactants compounding the membrane layer has the advantage of increasing the encapsulation efficiency and improving long-term stability, which enables the administration of a small amount of the nanocompound (45-47).

Liposomes (Figure 1, right) are spherical vesicles composed of a phospholipid bilayer and sterols (e.g., cholesterol) that are very similar to biological membranes and transport the active compound into an aqueous core (48-51). Liposomes are appropriate for the transportation of aqueous or lipid drugs. Their main advantage is that they are less degraded by enterocytes, thus increasing the bioavailability of hydrophobic compounds (32,52).

**Figure 2:**
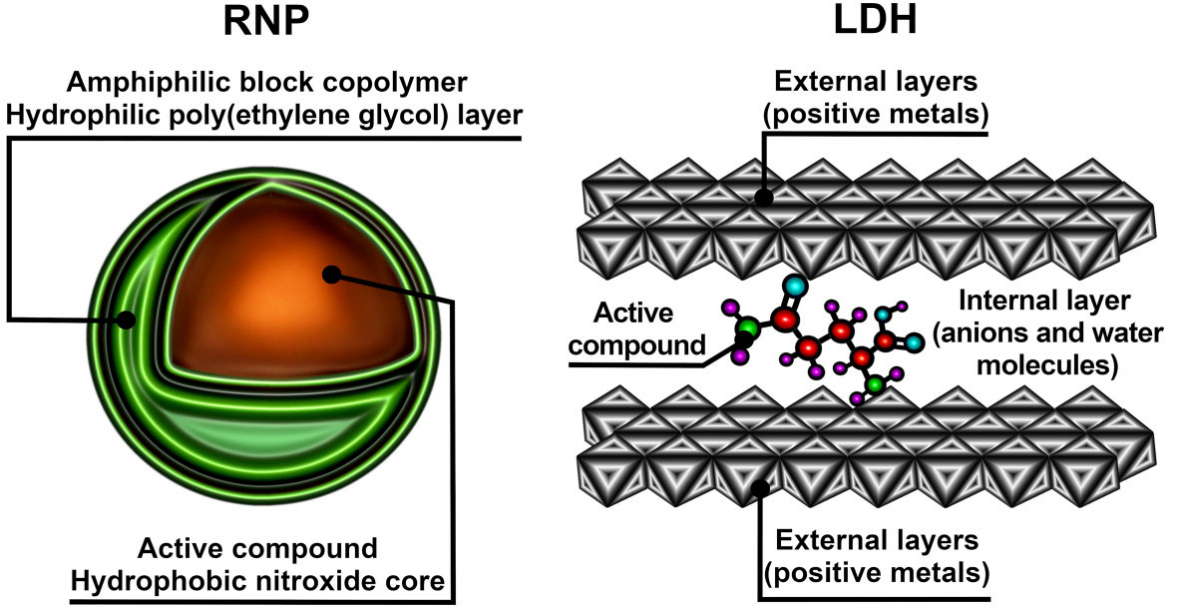
Schematic representation of nanocarriers redox-active nanoparticles (RNP) and layered double hydroxide nanoparticles (LDH).

Redox-active nanoparticles (Figure 2, left) are composed of amphiphilic block copolymers that form a hydrophilic poly(ethylene glycol) layer. The nitroxide radicals of the amphiphilic block copolymers are conjugated with hydrophobic segments, forming micelles with a hydrophobic nitroxide core in which the active compound is allocated (53). Redox-active nanoparticles are useful for the transportation of antioxidants as they protect the antioxidant in the core, decrease antioxidant toxicity, and reduce renal clearance, resulting in longer half-life blood circulation of the antioxidants in comparison to antioxidants in their free form (54,19).

Finally, layered double hydroxide nanoparticles (also called hydrotalcite-like material or anionic clays, Figure 2, right) consist of different metals with a positive charge in the external layers, while the internal layers are filled with anions and water molecules. The active compound is allocated between the external and internal layers. The most important advantage of layered double hydroxide nanoparticles is that their 2D structure provides a large surface area, which results in high drug loading efficiency and elevated bioactivity (55-58).

## Absorption of nutritional nanocompounds

**Figure 3:**
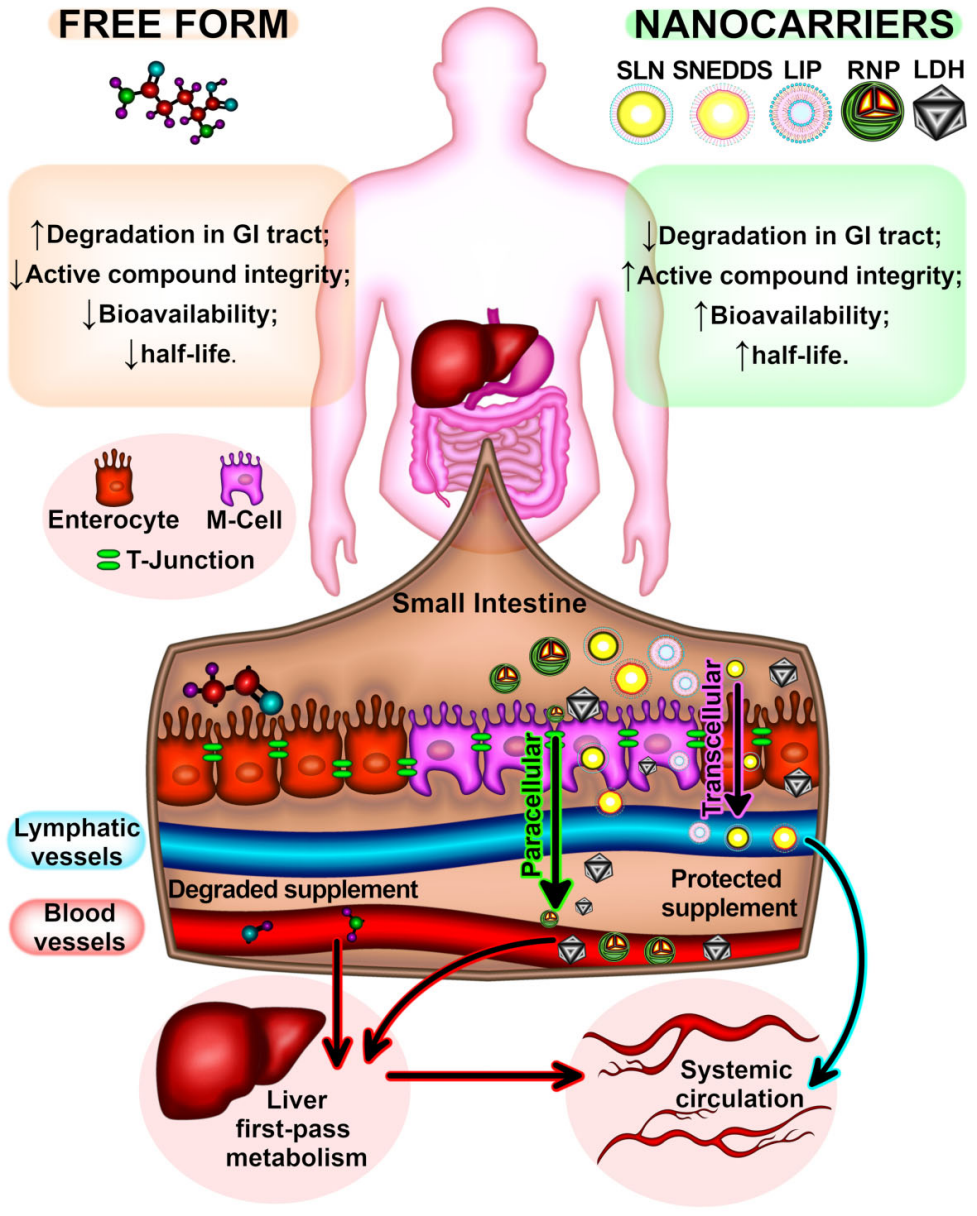
Advantages of nanoencapsulated active compounds in relation to non-nanoencapsulated active compounds during gastrointestinal (GI) transit and absorption. Solid lipid nanoparticles (SLN), self-nanoemulsifying drug delivery system (SNEDDS), and liposomes (LIP) are nanocarriers that are directly absorbed in the lymphatic vessels, thus free of liver first-pass metabolism. Redox-active nanoparticles (RNP) and layered double hydroxide nanoparticles (LDH) are absorbed in blood vessels, thus subjected to first-pass metabolism before entering in the systemic circulation.

A remarkable advantage of nanoencapsulated active compounds is their high absorption rate in the intestinal epithelial barrier (Figure 3). Some non-nanoencapsulated active compounds are greatly degraded by stomach acidity and gastrointestinal tract enzymes (59). In addition, after absorption, non-nanoencapsulated active compounds can be degraded in the first-pass metabolism (41,60). Consequently, non-nanoencapsulated active compounds have low bioavailability. In fact, a study reported that the bioavailability of the non-nanoencapsulated form is from 1.85- to 1.91-fold lower than that of the nanoencapsulated form (61).

Lipid nanosystems provide protection against pH changes and enzymatic actions in the gastrointestinal tract, which in turn preserves the integrity of the active compound (62). In addition, lipid nanosystems associate with lipoproteins in the small intestine, forming chylomicrons (41,63). The nanoparticle-chylomicron complex crosses the intestinal cell barrier by the transcytosis/transcellular or paracellular route between tight junctions (51,63). Because the nanoparticle-chylomicron complex is too large to enter blood capillaries, it is absorbed by the lymphatic vessels (64,65). Thus, nanoparticle-chylomicron complex does not enter in the first-pass metabolism of the liver (60,66), increasing the bioavailability of the active compound (67). In fact, another study reported that nanoparticles increase compound bioavailability by 3.2-fold compared with the non-nanoencapsulated form (14).

Redox-active nanoparticles and layered double hydroxide nanoparticles are also absorbed by transcellular or paracellular routes. However, as they do not have a lipid composition, they are absorbed in blood capillaries and pass through the liver before reaching the systemic circulation. While redox-active nanoparticles and layered double hydroxide nanoparticles do not protect from first-pass metabolism, they offer protection against pH and enzymatic actions in the gastrointestinal tract, increasing the bioavailability of the active compound. According to another study using redox-active nanoparticles to suppress inflammatory bowel disease, plasma concentration of silymarin was higher (5 µg/mL) when administered with redox nanoparticles than when administered with the non-nanoencapsulated form (1 µg/mL) (68).

## Nutritional nanocompounds and exercise performance

Only a few studies have tested the effectiveness of nanocompounds for the improvement of exercise performance. These studies used nanocomposites containing the following active compounds: resveratrol, iron, nitroxide radicals, and folic acid. The main findings of these studies are summarized in the following subsections and in Table 1.

**Table 1:** Summary of studies investigating the effect of nanocompounds on exercise performance.

Study	Animal model	Nanocarrier	Protocol	Performance test	Effects(compared to non-nanoencapsulated form)	Effects(compared to control/placebo)
**Resveratrol**						
Sun et al. 2019 36	C57BL/6J mice	SLN	Running training, 120 min/day, for 8 weeks + 25 mg/kg administered 1 h before exercise, 6 days/week, for 8 weeks	Time to exhaustion running test	No effect. However, only nanocompound presented changes compared to the control (see right column).	↑ Running distance; ↓ Respiratory exchange ratio during low-moderate intensity exercise; ↑ Mitochondrial biogenesis and elimination of dysfunctional mitochondria; ↑ Mitochondrial function.
Qin et al. 2020 37	C57BL/6J mice	SLN	Running training, 120 min/day, for 4 weeks + 150 mg/kg ingested once daily, 6 days/week, for 8 weeks	Time to exhaustion running test	No effect. However, only nanocompound presented changes compared to the placebo (see right column).	↑ Time to exhaustion and running distance; ↓ Lipid peroxidation and oxidative stress; ↓ Levels of malondialdehyde and lipid peroxidation; ↑ Levels of superoxide dismutase, glutathione peroxidase, and catalase; ↑ Muscular fiber integrity (presence of normal fiber shape); ↓ Inflammatory infiltration, edema, and myonecrosis.
Yen et al. 2017 14	Sprague-Dawley rats	SNEEDS	50 mg/kg administered 6 h before exercise	Time to exhaustion swimming test (load of 15% of body weight)	↑ Mean maximum concentration of resveratrol; ↑ Oral resveratrol bioavailability; ↑ Time to exhaustion.	↑ Time to exhaustion; ↓ Serum ammonia; ↑ Plasma glucose; ↑ Lactate clearance during exercise.
**Iron**						
Xu et al. 2014 38	Wistar rats	LIP	High intensity running training for 4 weeks to induce anemia. After anemia was confirmed, 2 more weeks of training receiving ferric ammonium citrate liposomes or heme iron liposomes at 5 mg iron/100 g body weight	No measurement of performance	↑ Serum iron; ↑ Red blood cells, hematocrit, and hemoglobin; ↑ Liver iron content and superoxide dismutase in heme iron liposomes *vs* free heme iron; ↑ Serum and liver superoxide dismutase in ferric ammonium citrate liposomes *vs* free ferric ammonium citrate; ↓ Serum and liver malondialdehyde superoxide dismutase in heme iron liposomes *vs* free heme iron.	-
**Nitroxide radicals**						
Toriumi et al. 2021 19	Fischer 344 rats	RNP	Subcutaneous infusion of redox-active nanoparticles (from 200 to 400 mg/kg) before exercise	Time to exhaustion running test at maximal aerobic velocity	↓ Time to exhaustion in low molecular weight free antioxidant supplementation; Presence of mitochondria damage in low molecular weight free antioxidant supplementation.	↑ Time to exhaustion in a dose-dependent manner; ↓ Hemolysis; ↓ Exercise-induced reduction in red blood cells and increase in plasma free iron; ↓ Oxidative stress in the skeletal muscle; ↓ Markers of muscle damage in the blood.
**Folic acid**						
Qin et al. 2014 81	Kunming mice	LDH	5 mg/kg for 28 days	Time to exhaustion swimming test (load of 10% of body weight)	Tendency to prolong the time to exhaustion, increase muscle and hepatic glycogen levels, and reduce blood urea nitrogen and lactic acid (non-significant).	↑ Time to exhaustion; ↑ Muscle and hepatic glycogen levels; ↓ Blood urea nitrogen and blood lactic acid.

SLN: Solid lipid nanoparticles; SNEDDS: self-nanoemulsifying drug delivery system; LIP: liposomes; RNP: Redox-active nanoparticles; LDH: layered double hydroxide nanoparticles.

### Resveratrol in lipid-based nanosystems

Resveratrol, a naturally occurring polyphenol (trans-3,4',5-trihydroxystilbene), presents anti-inflammatory, antitumorigenic, and antioxidant properties (68). While resveratrol might be useful to delay fatigue and increase exercise performance (69), the clinical application of resveratrol is still a challenge due to its low solubility and bioavailability (37,70). In this regard, solid lipid nanoparticles and the self-nanoemulsifying drug delivery system have been used to overcome these drawbacks and expand resveratrol applications (14,36).

One study evaluated C57BL/6J mice during an 8-week running training protocol with an initial speed of 10 m/min maintained for 120 min per day and gradually increased to 20 m/min, performed until exhaustion (36). Supplements were administered 1 h before exercise, 6 days per week, for 8 weeks (resveratrol-loaded solid lipid nanoparticles, free-form resveratrol, or control). After the training period, the average running distance to exhaustion was 28.7% longer in mice that ingested resveratrol-loaded solid lipid nanoparticles (8617.8 m) compared to mice of the control group (6695.3 m). However, it was not significantly different from mice that ingested free-form resveratrol (7364.5 m). In addition, the respiratory exchange ratio during low-moderate intensity exercise decreased with the ingestion of resveratrol-loaded solid lipid nanoparticles. As a reduction in the respiratory exchange ratio typically reflects a substrate shift favoring fat metabolism, the authors concluded that the ingestion of resveratrol-loaded solid lipid nanoparticles increased fat oxidation rate. It should be mentioned, however, that data of respiratory exchange ratio were only presented graphically, which precludes the quantification of the extent of the changes in fat oxidation rate. Finally, resveratrol-loaded solid lipid nanoparticles improved the balance between mitochondrial biogenesis and mitophagy, providing a precise control between the production of functional mitochondria and selective elimination of dysfunctional mitochondria, thereby improving mitochondrial function.

Another study evaluated the potential of resveratrol-loaded solid lipid nanoparticles on running performance of C57BL/6J mice (37). Running training was performed for 4 weeks, 120 min per day, and mice ingested resveratrol-loaded solid lipid nanoparticles, free-form resveratrol, or placebo once a day, 6 days per week, for 8 weeks (starting four weeks before the training). Time to exhaustion and running distance during a forced running capacity test were significantly longer in the group supplemented with resveratrol-loaded solid lipid nanoparticles, but not in the group supplemented with free-form resveratrol compared to the placebo group (data only presented graphically). Resveratrol-loaded solid lipid nanoparticles also reduced lipid peroxidation and oxidative stress and improved antioxidant defense (data only presented graphically). Muscular fiber integrity was also more preserved in the group supplemented with resveratrol-loaded solid lipid nanoparticles, presenting a higher incidence of fibers with normal shape and lower inflammatory infiltration, edema, and myonecrosis. Based on these findings, the authors concluded that resveratrol-loaded solid lipid nanoparticles might be useful in improving endurance capacity and facilitate recovery from exhaustive exercise.

In a more recent study with resveratrol, the bioavailability of resveratrol when administrated via a self-nanoemulsifying drug delivery system was compared with its non-nanoencapsulated form (14). Blood samples of Sprague-Dawley rats were regularly taken to evaluate the pharmacokinetics of the compound. The mean maximum concentration of resveratrol in blood was 2.2-fold higher when administered in nanoparticles than in its free form (869.2±112.2 and 386.2±68.4 ng/mL, respectively). The oral bioavailability of the resveratrol administered in nanoparticles was 9.5±1.5%, three times higher than in its free form (3.0±0.8%). When resveratrol was administrated via a self-nanoemulsifying drug delivery system, time to exhaustion during an exhaustive swimming test was 2.1-fold higher than in the vehicle group and 1.8-fold higher than in the resveratrol free form group. In addition, serum ammonia was lower and plasma glucose and lactate clearance were higher in the group treated with resveratrol via a self-nanoemulsifying drug delivery system compared with the vehicle group.

Thus, these findings suggest that the nanoencapsulation of resveratrol improves endurance performance in mice. This gain in endurance performance with the use of nanoencapsulated resveratrol has been mainly associated with: 1) improved antioxidant activity; 2) reduced lipid peroxidation; 3) muscle integrity preservation; 4) lower inflammation; 5) better mitochondrial function and quality; 6) increased fat oxidation and serum glucose levels; and 7) higher lactate clearance.

### Iron into liposomes

Exercise training might induce transitory anemia and iron storage deficiency resulting in increased symptoms of fatigue and impaired exercise performance (71,72). Thus, iron supplementation is widely used in the treatment of exercise-related anemia (73). The conventional form of iron supplementation is, however, correlated with gastrointestinal complications, such as constipation, bloating, intestinal mucosa inflammation (74), and lower intestinal absorption (75). Thus, nanoencapsulation of iron using liposomes is an alternative for improving absorption and avoiding oxidative stress and other side effects related to the conventional form of iron supplementation (50,75).

Only one study investigated the effects of iron carried by liposomes on exercise-induced anemia (38). In that study, Wistar rats performed a high-intensity running training for 4 weeks to induce anemia; thereafter, the rats with confirmed anemia exercised for an additional two weeks while receiving iron supplementation by intragastric administration of ferric ammonium citrate liposomes or heme iron liposomes. Control groups were administered with equivalent amounts of non-encapsulated ferric ammonium citrate or heme iron. Serum iron, red blood cells, hematocrits, and hemoglobin were all higher in the ferric ammonium citrate liposome and heme iron liposome groups than in the respective groups that received the non-encapsulated forms of the supplement. Liver iron levels were also higher in the heme iron liposome group than in the non-encapsulated heme iron group (data only reported graphically). These data suggest that ferric ammonium citrate liposome and heme iron liposome supplementation have the potential to attenuate iron deficiency and exercise-induced anemia. In addition, ferric ammonium citrate liposomes also increased superoxide dismutase in serum and liver, and reduced malondialdehyde in serum compared to the free form ferric ammonium citrate. Similarly, heme iron liposomes increased superoxide dismutase in liver and reduced malondialdehyde in serum and liver compared to the free form heme iron groups.

These above findings suggest that ferric ammonium citrate liposomes and heme iron liposomes also reduce oxidative stress. Unfortunately, endurance performance was not assessed in this study, but as there is a straight association between anemia and fatigue (19,71,72), it could be inferred that iron into liposome supplementation might attenuate any reduction in endurance performance associated with anemia. Therefore, studies investigating the potential of ammonium citrate liposomes and heme iron liposomes on exercise performance are necessary.

### Nitroxide radicals in redox-active nanoparticles

Only one study explored the potential of nitroxide radicals in nanoparticles on exercise performance (19). Nitroxides are stable free radicals that contain antioxidant properties (76,77). Male Fischer rats (n=344) performed a time-to-exhaustion treadmill running test at maximal aerobic velocity after subcutaneous infusion of redox-active nanoparticles containing nitroxide radicals in the solid core (19). Compared to the control (62±6 min), the redox-active nanoparticles containing nitroxide radicals improved endurance performance (97±5 min) in a dose-dependent manner (from 200 to 400 mg/kg). In contrast, the supplementation with low molecular weight free antioxidant reduced endurance performance (48±2 min), especially at the highest dose (0.69 mmol/kg). Additionally, compared to the control group, redox-active nanoparticles containing nitroxide radicals restricted the exercise-induced reduction in the number of red blood cells and exercise-induced increase in plasma free iron and decreased oxidative stress in skeletal muscle and markers of muscle damage in blood (data only reported graphically). In addition, mitochondria damage was observed in the group treated with low molecular weight free antioxidant but not in the group treated with redox-active nanoparticles containing nitroxide radicals. These findings provide the first evidence that redox-active nanoparticles containing nitroxide radicals can improve endurance performance and reduce oxidative stress and mitochondria damage.

### Folic acid into layered double hydroxide nanoparticles

Folic acid is the synthetic form of folate (vitamin B9), an essential vitamin for maintaining erythropoiesis, nucleotide synthesis, and amino acid metabolism (78). Folic acid is also a potent antioxidant (79) and its deficiency can intensify symptoms of anemia and fatigue (80). However, the effectivity of folic acid supplementation is limited due to its poor stability, short half-life, and low bioavailability (81). Thus, nanoencapsulation is a promising strategy to improve the effectivity of folic acid supplementation. To our knowledge, however, only one study has explored the potential of nanoencapsulated folic acid supplementation on exercise performance (81).

The potential of encapsulating folic acid into layered double hydroxide nanoparticles to improve exercise performance and antioxidant defense was evaluated in Kunming mice (81). Mice were supplemented for 28 days with free folic acid, folic acid into layered hydroxide nanoparticles, or placebo (distilled water). Compared to the free folic acid, supplementation with folic acid into layered hydroxide nanoparticles tended to prolong the time to exhaustion during a swimming test to 13.12 min (51% longer compared with the control group), increase muscle and hepatic glycogen levels (data only reported graphically), and reduce blood urea nitrogen and lactic acid by 19.1 and 30.5%, respectively, compared with the control group. However, these differences did not reach statistical significance, probably due to the low number of mice in each group (n=3). In addition, folic acid into layered hydroxide nanoparticles showed no significant toxicity to normal cells.

## Limitations and future directions

While research using nanotechnology is growing and many studies have explored the potential of several nanoencapsulated active compounds on health and disease treatment (82,83), a limited number of studies have been conducted in the context of exercise performance. The present review explored the main findings of studies applying nutritional nanocompounds for exercise performance, which, in general, indicate the effectiveness of nano-supplements for improving exercise performance. However, some limitations of these studies should be addressed. First, one study (81) was conducted with a very small sample size (n=3), which precludes appropriate statistical inference. Second, some studies (36,37) demonstrated positive effects of the nanoencapsulated supplement when compared with a control/placebo group, but these positive effects were not found when compared with the group that received the compound in its free form (i.e., non-nanoencapsulated from). Furthermore, in some studies (14,81) the statistical comparisons between nanoencapsulated supplement *vs* non-nanoencapsulated supplement were not clearly reported, which raises the question of the true effectiveness of the nanocompound. Third, there is a small number of studies for each nanoencapsulated supplement; thus, it is clear that more studies are necessary to expand the findings. Finally, no studies have evaluated oral nano-supplementation in humans. While nanocompounds do not appear to be toxic (81), future studies should apply this new form of supplementation in humans to evaluate possible adverse effects for the safe use of nano-supplements for improving exercise performance.

Another important issue is that studies included in the present review encompassed a limited scope of active compounds. Since nutritional supplements are frequently used to improve exercise performance, such as creatine, caffeine, beta-alanine, sodium bicarbonate, and nitrate, there is an open avenue for future studies to evaluate the efficacy of nanoencapsulation of other nutritional supplements for exercise performance improvement. Finally, the present review focused on nutritional supplementation, but there are also other possible forms of application of nanotechnology to improve exercise performance, such as transdermal, for example (84).

## Conclusion

The present review indicated the potential effectivity of nano-supplementation to improve exercise performance. Positive physiological effects related to exercise performance, such as increased antioxidant activity, reduced inflammatory status, maintenance of cellular integrity of muscle and red blood cells, improved mitochondrial function and quality, improved energy substrate balance, and increased serum and liver iron content were also reported with the use of nano-supplementation. Thus, nanocompounds have promising potential to promote improvement in exercise and sports performance.
